# State-of-the-Art Statistical Approaches for Estimating Flood Events

**DOI:** 10.3390/e24070898

**Published:** 2022-06-29

**Authors:** Muhammad Fawad, Felício Cassalho, Jingli Ren, Lu Chen, Ting Yan

**Affiliations:** 1 Zhengzhou Key Laboratory of Big Data Analysis and Application, Henan Academy of Big Data, Zhengzhou University, Zhengzhou 450052, China; fawadkhn42@gmail.com (M.F.); renjl@zzu.edu.cn (J.R.); 2 School of Mathematics and Statistics, Zhengzhou University, Zhengzhou 450001, China; 3 Department of Civil, Environmental, and Infrastructure Engineering, George Mason University, 4400 University Dr, Fairfax, VA 22030, USA; fcassalh@gmu.edu; 4 College of Hydropower & Information Engineering, Huazhong University of Science & Technology, Wuhan 430074, China; chen_lu@hust.edu.cn; 5 Department of Statistics, School of Mathematics and Statistics, Central China Normal University, Wuhan 430079, China

**Keywords:** water resources management, extreme events, return periods, probability distributions, nonparametric

## Abstract

Reliable quantile estimates of annual peak flow discharges (APFDs) are needed for the design and operation of major hydraulic infrastructures and for more general flood risk management and planning. In the present study, linear higher order-moments (LH-moments) and nonparametric kernel functions were applied to APFDs at 18 stream gauge stations in Punjab, Pakistan. The main purpose of this study was to evaluate the impacts of different quantile estimation methods towards water resources management and engineering applications by means of comparing the state-of-the-art approaches and their quantile estimates calculated from LH-moments and nonparametric kernel functions. The LH-moments (η = 0, 1, 2) were calculated for the three best-fitted distributions, namely, generalized logistic (GLO), generalized extreme value (GEV), and generalized Pareto (GPA), and the performances of these distributions for each level of LH-moments (η = 0, 1, 2) were compared in terms of Anderson–Darling, Kolmogorov–Smirnov, and Cramér–Von Mises tests and LH-moment ratio diagrams. The findings indicated that GPA and GEV distributions were best fitted for most stations, followed by GLO distribution. The quantile estimates derived from LH-moments (η = 0, 1, 2) had a lower relative absolute error, particularly for higher return periods. However, the Gaussian kernel function provided a close estimate among nonparametric kernel functions for small return periods when compared to LH-moments (η = 0, 1, 2), thus highlighting the importance of using LH-moments (η = 0, 1, 2) and nonparametric kernel functions in water resources management and engineering projects.

## 1. Introduction 

Extreme environmental events have always been a vital part of human history. Floods, rainstorms, droughts, and windstorms are some of the manifestations of these events, which cause enormous destruction. Flood is often referred to as one of the most devastating natural disasters in terms of damage to property, infrastructure, and the environment, even threatening human lives. 

Quantile estimations of floods, commonly extracted from annual peak flow discharges (APFDs), are of great importance for the description of such events. These estimates allow for the assessment of flood characteristics by associating their magnitude to a corresponding frequency, from which mitigating hydraulic structures and management practices may be designed. The objective of flood frequency analysis (FFA) is to obtain a flood quantile magnitude estimator for one or more stations on the river system. Depending on the magnitude of the flood, an estimate of its return period may be required. The common interest in estimating quantiles of extreme floods for different return periods, i.e., 50-year, 100-year, or 500-year flood, is the design of hydraulic structures such as dams, culverts, and bridges [1,2,3].

The parametric estimation method (LH-moments) and nonparametric method (kernel functions) were used in the present study to estimate the flood magnitude at different return periods. In parametric methods, the APFDs are assumed to be independent and identically distributed, as well as being drawn from a population with a known probability distribution function (PDF). In addition, an adequate PDF is selected from a set of candidate PDFs using a robust goodness-of-fit test. As described by [4], extensive literature for FFA is available, not only for the detailed description of PDFs, but also for parameter estimation. The commonly used PDFs for modeling of the APFDs include: generalized extreme value (GEV), generalized logistic (GLO), generalized Pareto (GPA), Pearson type 3 (P3), log Pearson type 3 (LP3), Weibull (WEI), extreme value type 1, extreme value type 2, normal, log normal, gamma, and exponential [4,5,6,7,8,9]. 

The widely used parameter estimation techniques for PDFs include maximum likelihood, L-moments, LH-moments, and method of moments. The main drawback of the maximum likelihood and method of moments is that the product moments of the APFDs are similarly affected by the small sample size. Furthermore, the higher moments (e.g., coefficient of variation and skewness) are greatly influenced by the extremes observation in the data series [6]. On the other hand, L-moments are less affected by extreme observations in the data series [10]. Wang [11] introduced LH-moments, which are the generalization of L-moments, using higher order, i.e., “H”, L-moments. Wang found that LH-moments produce consistent quantile estimates for large return periods, since LH-moments provide greater weight to the larger values in the APFD series and hence better fits to the upper tail of PDFs. This characteristic is even more relevant when sample sizes are small, which is a common reality of streamflow monitoring in developing countries, including Pakistan [8,9,12,13,14]. Various studies have been conducted on LH-moments in different regions of the world [11,15,16,17,18,19,20,21,22,23]. Most of these researchers have focused on comparing LH-moments with L-moments by using goodness-of-fit-tests for different PDFs.

The aforementioned studies were based on parametric methods, which require a priori PDF selection. Alternative nonparametric methods do not assume the APFD series in a distributional form. Many studies on FFA based on the nonparametric approach have been conducted [24,25,26,27,28,29,30,31,32,33], among which kernel function estimators have stood out for producing the most reliable nonparametric methods. Adamowski [24,25] proposed a nonparametric kernel estimation for FFA and conducted a Monte-Carlo simulation experiment in order to compare the nonparametric approach with parametric PDFs, namely, LP3 and WEI distributions. His results show that the nonparametric method produces more accurate estimates than parametric methods, but the probability of extrapolation is lower than the highest data observed in the sample. Lall et al. [34] stressed the choice of a kernel function that reflects the shape and bandwidth of FFA. Kernel function and bandwidth selection techniques are implemented in three situations: Gaussian data, skewed data, and mixed data. 

By definition, the extreme event is rare and often occurs in a short time period; therefore, estimating floods for large return periods is a challenging task, often leading to gross errors in the estimation of quantiles. Another problem is the identification of a suitable statistical model. The standard methods of estimation, including maximum likelihood, method of moments, least squares, may not give consistent quantile estimates for large return periods when the sample size is small. Therefore, we need an appropriate method of estimation that gives consistent quantile estimates [35]. The current research attempts to highlight the significance of LH-moments (η = 0, 1, 2) and nonparametric kernel functions in FFA. On the one hand, this allows for comparison of the two estimation approaches within this framework, while on the other hand, it observes the effect of utilizing RAE to evaluate quantiles for various return periods. This study does not imply that nonparametric kernel functions are always the best fit and should be used in place of adequately constructed parametric methods. However, the possibility of obtaining the best fit for nonparametric kernel functions may be more significant when using FFA, as these approaches give precise estimations for observed flood variables [36]. Therefore, the current study emphasizes the significance of using LH-moments (η = 0, 1, 2) and nonparametric kernel functions to develop a comprehensive framework for at-site FFA. The steps in the framework are as follows: (i) selection of the optimized PDFs with a comprehensive level of LH-moments (η = 0, 1, 2) for the annual peak flow of 18 stations; (ii) estimation of quantiles for various flood return periods through LH-moments (η = 0, 1, 2) and nonparametric kernel functions; and (iii) comparison of various flood return periods in terms of RAE. This paper is arranged into five sections. Section 2 contains a detailed step-by-step explanation of the methodology. The details of the study area are presented in Section 3. The results and discussions of the study are provided in Section 4. Section 5 summarizes the conclusions of the study.

## 2. Methods

### 2.1. Linear Higher Order-Moments (LH-Moments)

LH-moments were proposed by [11] as the expectations of a linear combination of higher-order statistics. Let *n* be the sample size drawn from the distribution F(x)=Pr(X≤x); then the four LH-moments are defined as follows:

(1) $$\lambda_{1}^{\eta }=E\left[X_{\left(\eta +1\right):\left(\eta +1\right)}\right]$$

(2) $$\lambda_{2}^{\eta }=\frac{1}{2}E\left[X_{\left(\eta +2\right):\left(\eta +2\right)}-X_{\left(\eta +1\right):\left(\eta +2\right)}\right]$$

(3) $$\lambda_{3}^{\eta }=\frac{1}{3}E\left[X_{\left(\eta +3\right):\left(\eta +3\right)}-2X_{\left(\eta +2\right):\left(\eta +3\right)}+X_{\left(\eta +1\right):\left(\eta +3\right)}\right]$$

(4) $$\lambda_{4}^{\eta }=\frac{1}{4}E\left[X_{\left(\eta +4\right):\left(\eta +4\right)}-3X_{\left(\eta +3\right):\left(\eta +4\right)}+3X_{\left(\eta +2\right):\left(\eta +4\right)}-X_{\left(\eta +1\right):\left(\eta +4\right)}\right]$$

where

(5) $$E\left[X_{\left(j:n\right)}\right]=\frac{m!}{\left(j-1\right)!\left(m-1\right)!}\overset{1}{\underset{0}{\int }}x\left(F\right)\;F^{j-1}{\left(1-F\right)}^{n-j}dF$$

When η = 0, LH-moments become equal to L-moments [10]. As η increases, it reflects more and more characteristics of the upper part of the PDFs and extreme events in data. Here, $\lambda_{1}^{\eta }$ provides the location of the distribution, $\lambda_{2}^{\eta }$ is the spreadness of the distribution,$\;\lambda_{3}^{\eta }$ represents how the upper part of the distribution is asymmetric, and $\lambda_{4}^{\eta }$ measures the peakedness of the upper parts of the distributions. For η = 0, 1, 2, the LH-moments are referred to as L-moments, L1-moments, and L2-moments, respectively. The LH-moment ratios are described below.

(6) $$\tau_{2}^{\eta }=\frac{\lambda_{2}^{\eta }}{\lambda_{1}^{\eta }}$$

(7) $$\tau_{3}^{\eta }=\frac{\lambda_{3}^{\eta }}{\lambda_{2}^{\eta }}$$

(8) $$\tau_{4}^{\eta }=\frac{\lambda_{4}^{\eta }}{\lambda_{2}^{\eta }}$$

Let $x_{1}\leq x_{2}\leq \ldots \leq x_{n}$ be the order statistic; then the unbiased estimators of LH-moments are given below.

(9) $${\hat{\lambda }}_{1}^{\eta }=\frac{1}{\left(\begin{array}{c} n\\ \eta +1 \end{array}\right)}\overset{n}{\underset{i=1}{\sum }}\left(\begin{array}{c} i-1\\ \eta \end{array}\right)x_{i}$$

(10) $${\hat{\lambda }}_{2}^{\eta }=\frac{1}{2\left(\begin{array}{c} n\\ \eta +1 \end{array}\right)}\overset{n}{\underset{i=1}{\sum }}\left[\left(\begin{array}{c} i-1\\ \eta +1 \end{array}\right)-\left(\begin{array}{c} i-1\\ \eta \end{array}\right)\left(\begin{array}{c} n-i\\ 1 \end{array}\right)\right]x_{i}$$

(11) $${\hat{\lambda }}_{3}^{\eta }=\frac{1}{3\left(\begin{array}{c} n\\ \eta +1 \end{array}\right)}\overset{n}{\underset{i=1}{\sum }}\left[\left(\begin{array}{c} i-1\\ \eta +2 \end{array}\right)-2\left(\begin{array}{c} i-1\\ \eta +1 \end{array}\right)\left(\begin{array}{c} n-1\\ 1 \end{array}\right)+\left(\begin{array}{c} i-1\\ \eta \end{array}\right)\left(\begin{array}{c} n-i\\ 2 \end{array}\right)\right]x_{i}$$

(12) $${\hat{\lambda }}_{4}^{\eta }=\frac{1}{4\left(\begin{array}{c} n\\ \eta +1 \end{array}\right)}\overset{n}{\underset{i=1}{\sum }}\left[\left(\begin{array}{c} i-1\\ \eta +3 \end{array}\right)-3\left(\begin{array}{c} i-1\\ \eta +2 \end{array}\right)\left(\begin{array}{c} n-i\\ 1 \end{array}\right)+3\left(\begin{array}{c} i-1\\ \eta +1 \end{array}\right)\left(\begin{array}{c} n-i\\ 2 \end{array}\right)-\left(\begin{array}{c} i-1\\ \eta \end{array}\right)\left(\begin{array}{c} n-i\\ 3 \end{array}\right)\right]x_{i}$$

The sample LH-moment ratios are as follows:

(13) $${\hat{\tau }}_{2}^{\eta }=\frac{{\hat{\lambda }}_{2}^{\eta }}{{\hat{\lambda }}_{1}^{\eta }}$$

(14) $${\hat{\tau }}_{3}^{\eta }=\frac{{\hat{\lambda }}_{3}^{\eta }}{{\hat{\lambda }}_{2}^{\eta }}\;$$

(15) $${\hat{\tau }}_{4}^{\eta }=\frac{{\hat{\lambda }}_{4}^{\eta }}{{\hat{\lambda }}_{2}^{\eta }}$$

### 2.2. Estimation of the Parameters of the Selected PDFs Based on LH-Moments

Several PDFs for fitting flood series exist in FFA; among them, the GLO, GEV, and GPA distributions have been recommended in Pakistan by various researchers to model extreme flood events [8,9,13,14,37,38,39]. Based on the comprehensive literature, three PDFs were selected in the current study, i.e., GLO, GEV, and GPA. The parameters of each respective PDF were estimated using the LH-moments and given below.

#### 2.2.1. Generalized Logistics (GLO) Distribution 

GLO distribution is a generalized variant of the logistic distribution [40,41] that has been applied in recent years to assess extreme value events. Since GLO was recognized as the acceptable approach for FFA in the UK [42], its use in hydrology has gained popularity [43]. The PDF, distribution function (DF), and quantile function (QF) of the GLO distribution are expressed, respectively, by:

(16) $$f\left(x\right)=\frac{1}{\alpha }{\left[1-k\left(\frac{x-\xi }{\alpha }\right)\right]}^{\left(\frac{1}{k}-1\right)}{\left[1+{\left\{1-k\left(\frac{x-\xi }{\alpha }\right)\right\}}^{\left(\frac{1}{k}\right)}\right]}^{-2}$$

(17) $$F\left(x\right)={\left[1+{\left\{1-k\left(\frac{x-\xi }{\alpha }\right)\right\}}^{\left(\frac{1}{k}\right)}\right]}^{-1}$$

(18) $$x\left(F\right)=\xi +\frac{\alpha }{k}\left[1-{\left(\frac{1-F}{F}\right)}^{k}\right]$$

where *x* is the APFDs, *k* is the shape parameter, *ξ* is a location, and *α* is a scale parameter. These parameters are estimated by [17] using LH-moments and given below:

(19) $$\hat{\alpha }=\frac{\Gamma \left(\eta +2\right)\left(\eta +2\right){\hat{\beta }}_{n+1}-\left(\eta +1\right){\hat{\beta }}_{n}}{\Gamma \left(\eta +1-\hat{k}\right)\Gamma \left(1+\hat{k}\right)}$$

(20) $$\hat{\xi }=\left(\eta +1\right){\hat{\beta }}_{n}-\frac{\hat{\alpha }}{\hat{k}}\left[1-\frac{\Gamma \left(\eta +1-\hat{k}\right)\Gamma \left(1+\hat{k}\right)}{\Gamma \left(\eta +1\right)}\right]$$

(21) $$\hat{k}=-\frac{\left(\eta +3\right)\left(\eta +2\right){\hat{\beta }}_{n+2}-\left[{\left(\eta +2\right)}^{2}+\left(\eta +2\right)\left(\eta +1\right)\right]{\hat{\beta }}_{n+1}+{\left(\eta +1\right)}^{2}{\hat{\beta }}_{n}}{\left(\eta +2\right){\hat{\beta }}_{n+1}-\left(\eta +1\right){\hat{\beta }}_{n}}$$

#### 2.2.2. Generalized Extreme Value (GEV) Distribution 

The GEV was developed by [44] as a feasible tool for extreme value analysis, and it has gained widespread favor in FFA. The GEV distribution PDF, DF, and QF are written, respectively, as:

(22) $$f\left(x\right)=\frac{1}{\alpha }{\left[1-k\left(\frac{x-\xi }{\alpha }\right)\right]}^{\left(\frac{1}{k}-1\right)}e^{-{\left[1-k\left(\frac{x-\xi }{\alpha }\right)\right]}^{\left(\frac{1}{k}\right)}}$$

(23) $$F\left(x\right)=exp\left\{-{\left[1-k\left(\frac{x-\xi }{\alpha }\right)\right]}^{\frac{1}{k}}\right\}$$

(24) $$x\left(F\right)=\xi +\frac{\alpha }{k}\left[1-{\left(-lnF\right)}^{k}\right]$$

where *k*, *ξ*, *α* are shape, location, and scale parameters, respectively, which are estimated by [11] and are described below:

(25) $$\hat{\alpha }=\frac{\hat{k}\left[\left(\eta +2\right){\hat{\beta }}_{n+1}-\left(\eta +1\right){\hat{\beta }}_{n}\right]}{\Gamma \left(1+\hat{k}\right)\left[{\left(\eta +1\right)}^{-\hat{k}}-{\left(\eta +2\right)}^{-\hat{k}}\right]}$$

(26) $$\hat{\xi }=\left(\eta +1\right){\hat{\beta }}_{n}-\frac{\hat{\alpha }}{\hat{k}}\left[1-{\left(\eta +1\right)}^{-\hat{k}}\Gamma \left(1+\hat{k}\right)\right]$$

(27) $$\hat{k}=a_{0}+a_{1}\left[\tau_{3}^{\eta }\right]+a_{2}{\left[\tau_{3}^{\eta }\right]}^{2}+a_{3}{\left[\tau_{3}^{\eta }\right]}^{3}$$

#### 2.2.3. Generalized Pareto (GPA) Distribution 

Pickands [45] proposed GPA distribution, and several scholars have widely acknowledged it as the logical alternative for evaluating severe events [46,47]. The PDF, DF, and QF for GPA distribution are given, respectively, by:

(28) $$f\left(x\right)=\frac{1}{\alpha }{\left[1-\frac{k}{\alpha }\;\left(x-\xi \right)\right]}^{\left(\frac{1}{k}-1\right)}$$

(29) $$F\left(x\right)=1-{\left[1-\frac{k}{\alpha }\;\left(x-\xi \right)\right]}^{\frac{1}{k}}$$

(30) $$x\left(F\right)=\xi +\frac{\alpha }{k}\left[1-{\left(1-F\right)}^{k}\right]$$

The location (*α*), scale (*ξ*), and shape (*k*) parameters were estimated by [17] and are stated below:

(31) $$\hat{\alpha }=-\frac{\hat{k}\Gamma \left(\eta +3+\hat{k}\right)\Gamma \left(\eta +2+\hat{k}\right)\left(\eta +2\right){\hat{\beta }}_{\eta +1}-\left(\eta +1\right){\hat{\beta }}_{\eta }}{\left(\eta +1\right)!\Gamma \left(1+\hat{k}\right)\left[\left(\eta +2\right)\Gamma \left(\eta +2+\hat{k}\right)-\Gamma \left(\eta +3+\hat{k}\right)\right]}$$

(32) $$\hat{\xi }=\left(\eta +1\right){\hat{\beta }}_{n}-\frac{\hat{\alpha }}{\hat{k}}\left[1-\frac{\left(\eta +1\right)\Gamma \left(\eta +1\right)\Gamma \left(1+\hat{k}\right)}{\Gamma \left(\eta +2+\hat{k}\right)}\right]$$

(33) $$\hat{k}=-\frac{-5-2\eta +\frac{\left(\eta +3\right)\left(\eta +3\right){\hat{\beta }}_{\eta +2}-\left(\eta +1\right){\hat{\beta }}_{\eta }}{\left(\eta +2\right){\hat{\beta }}_{\eta +1}-\left(\eta +1\right){\hat{\beta }}_{\eta }}}{-1+\frac{\left(\eta +3\right){\hat{\beta }}_{\eta +2}-\left(\eta +1\right){\hat{\beta }}_{\eta }}{\left(\eta +2\right){\hat{\beta }}_{\eta +1}-\left(\eta +1\right){\hat{\beta }}_{\eta }}}$$

### 2.3. Goodness-of-Fit (GOF) Tests

Four statistical measures, namely, the Anderson–Darling (AD) test, Kolmogorov–Smirnov (KS) test, Cramér–Von Mises (CVM) test, and LH-moment ratios (LH-ratios) diagram, were used in this study to determine the GOF tests for the selection of PDFs using LH-moments (η = 0, 1, 2). The PDFs for the APFDs that produced the smallest values for all of these GOF measures (AD test, KS test, and CVM test) were determined as a best-fit, and hence they were chosen for further estimation of quantiles. These GOF tests were previously applied to peak flow data and are frequently used to choose the best-fitting PDFs in FFA [8,9,35,48].

The AD test is used to evaluate the fit of an observed distribution function (DF) to its theoretical DF. The AD test gives a higher weight to the PDF’s tail, which is a necessary feature in modeling extreme events [35,48]. Heo et al. [49] describe the AD test statistic as follows:

(34) $$A^{2}=-n-\frac{1}{n}\overset{n}{\underset{i=1}{\sum }}\left(2i-1\right)log\;F\left(x_{i}\right)-\frac{1}{n}\overset{n}{\underset{i=1}{\sum }}\left(2n-2i-1\right)log\;F\left(x_{i}\right)$$

Here, *A^2^* denotes the test result, *n* represents the sample size, *x* is the variable being studied, and $F\left(x_{i}\right)$ denotes the DF. 

The KS test is based on the empirical DF and is used to assess whether a sample is drawn from a hypothesized continuous distribution. Assuming we have a random sample $\left(x_{1},x_{2},x_{3},\ldots ,x_{n}\right)$ from some distribution, then empirical DF is as follows:

(35) $$F_{n}\left(x\right)=\frac{1}{n}\left[Number\;of\;observations\leq x\right]$$

The maximum vertical distance between the theoretical PDF and empirical DF determines the KS statistic (*D*). The KS test statistic (*D*) is as follows:

(36) $$D=max_{1\leq i\leq n}\left[F\left(x_{i}\right)-\frac{i-1}{n},\frac{i}{n}-F\left(x_{i}\right)\right]$$

where *x_i_* is the ith order statistic, *n* signifies the size of the random sample, and $F\left(x_{i}\right)$ indicates the theoretical DF.

The CVM test, an alternative to the KS test, is used to compare DF to a given empirical DF. Let $\left(x_{1:n}\leq x_{i:n}\leq x_{n:n}\right)$ be the order statistics of a sample size *n*; then the CVM test is suggested in [50]:

(37) $$\omega^{2}=\frac{1}{12n}+\overset{n}{\underset{i=1}{\sum }}\left[\frac{2i-1}{2n}-F\left(x_{i}\right)\right]$$

Hosking [10] initially proposed the L-moment ratio diagram as the simplest way to determine the best-fitted distribution for the actual data. The L-moment ratio diagram is extended to each level of LH-moments (η = 0, 1, 2) [11]. The LH-ratio diagram is based on the relationships between LH-skewness and LH-kurtosis. Therefore, this allows better discrimination between the PDFs, and hence the identification of parent distribution can also be achieved. 

### 2.4. Quantile Estimates for Different Return Periods of Floods Based on LH-Moments 

Various scientific fields are interested in estimating quantiles corresponding to different return periods. The return period is also known as a recurrence interval, defined as the average of inter-event times between flood events [4]. Sometimes, the hydrologist wants to know the chances of a flood reaching or exceeding a specific magnitude over a set time period. This is known as the probability of occurrence or the probability of exceedance. The probability that the exceedance for a given flood (*q*) with a return period (*T*) may be exceeded once in *T* years is computed as follows:

(38) $$\;P\left(Q_{T}\leq q\right)=\frac{1}{T}$$

Equation (39) shows the cumulative probability of non-exceedance as follows:

(39) $$F\left(Q_{T}\right)=P\left(Q_{T}\leq q\right)=1-P\left(Q_{T}>q\right)=1-\frac{1}{T}$$

Equation (39) is used to calculate the magnitude of a flood for given return periods. We can obtain quantile estimates for different return periods by substituting [*F(Q_T_) = 1 − 1/T*] in the quantile function of the GLO, GEV, and GPA distributions, as described below.

(40) $$\mathrm{GLO}\Rightarrow {\hat{x}}_{T}=\hat{\xi }+\frac{\hat{\alpha }}{\hat{k}}\left[1-{\left(T-1\right)}^{-\hat{k}}\right]$$

(41) $$\mathrm{GEV}\Rightarrow {\hat{x}}_{T}=\hat{\xi }+\frac{\hat{\alpha }}{\hat{k}}\left[1-{\left\{-log\left(1-\frac{1}{T}\right)\right\}}^{\hat{k}}\right]$$

(42) $$\mathrm{GPA}\Rightarrow {\hat{x}}_{T}=\hat{\xi }+\frac{\hat{\alpha }}{\hat{k}}\left[1-T^{-\hat{k}}\right]$$

Equations (40)–(42) above are used to calculate the quantile associated with the required return periods for the GLO, GEV, and GPA distributions at different levels of LH-moments (η = 0, 1, 2).

### 2.5. Quantile Estimates for Different Return Periods of Floods Based on Nonparametric Kernel Function 

The nonparametric kernel function is based on kernel smoothing of the empirical QF of the variable under study [32]. Let $\left(x_{1},\;x_{2},x_{3},\ldots ,\;x_{n}\right)$ be the series of observed APFDs arranged in ascending order; then the mathematical form of the kernel estimator of the nonparametric kernel function is expressed as [51]:

(43) $${\hat{f}}_{h}\left(x\right)=\frac{1}{nh}\overset{n}{\underset{i=1}{\sum }}K\left(\frac{x-x_{i}}{h}\right)$$

where *K*(.) refers to the kernel function prescribed type (Epanechnikov, Gaussian, Biweight, or Triweight), *n* denotes the observation’s sample size, and *h* represents the bandwidth or smoothing parameter that controls the variance of the nonparametric kernel function [25,34,52]. The relation between DF and density of the nonparametric kernel function is as follows:

(44) $${\hat{F}}_{h}\left(x\right)=\overset{x}{\underset{-\infty }{\int }}{\hat{f}}_{h}\left(t\right)dt=\frac{1}{n}\overset{n}{\underset{i=1}{\sum }}H\left(\frac{x-x_{i}}{h}\right)$$

where

$$H\left(x\right)=\overset{x}{\underset{-\infty }{\int }}K\left(t\right)dt$$

Equation (44) is widely used in hydrology to calculate the quantiles associated with various return periods. The quantiles obtained by using a nonparametric kernel distribution estimator are as follows (for details, see [53,54]):

(45) $${\hat{x}}_{T}={\hat{F}}_{h}^{-1}\left(1-\frac{1}{T}\right)$$

Estimating the nonparametric kernel density method necessitates the selection of a kernel function, *K*(.), and the computation of a smoothing parameter, or bandwidth, *h* (as shown in Equation (44). The choice of *K*(.) is less critical, and different types of kernel functions that provide good results can be used. This study applied the Epanechnikov, Gaussian, Biweight, and Triweight kernel functions, which are commonly used in the literature [24,25,53,54,55,56,57]. The expressions of standard kernel functions are given below:

(46) $$\mathrm{Epanechnikov}\Rightarrow k\left(x\right)=\{\begin{array}{c} \frac{3}{4}\left(1-x^{2}\right);\;\;\;\;\;\;\;\;\;if\;\left|x\right|\leq 1\\ 0;\;\;\;\;\;\;\;\;\;\;\;\;\;\;\;\;\;\;\;\;\;\;\;\;\;\;\;\;\;otherwise \end{array}$$

(47) $$\mathrm{Gaussian}\Rightarrow k\left(x\right)=\frac{1}{\sqrt{2\pi }}e^{\left(-\frac{x^{2}}{2}\right)};\;-\infty \leq x\leq \infty$$

(48) $$\mathrm{Biweight}\Rightarrow k\left(x\right)=\{\begin{array}{c} \frac{15}{16}{\left(1-x^{2}\right)}^{2};\;\;\;\;\;\;if\;\left|x\right|\leq 1\\ 0;\;\;\;\;\;\;\;\;\;\;\;\;\;\;\;\;\;\;\;\;\;\;\;\;\;otherwise \end{array}$$

(49) $$\mathrm{Triweight}\Rightarrow k\left(x\right)=\{\begin{array}{c} \frac{35}{32}{\left(1-x^{2}\right)}^{3};\;\;\;\;\;\;if\;\left|x\right|\leq 1\\ 0;\;\;\;\;\;\;\;\;\;\;\;\;\;\;\;\;\;\;\;\;\;\;\;\;\;otherwise \end{array}$$

In Equation (44), the smoothing parameter *h* plays a crucial role in the kernel estimator. In practice, selecting an effective technique for computing *h* for an observed data sample is a more complicated task due to the influence of the bandwidth on the shape of the associated estimate. If the value of *h* is small, we will obtain an undersmoothed estimator with a large variation. On the other hand, if *h* is large, the resultant estimator will be extremely smooth and will be farther away from the function that we are attempting to estimate [32,53]. In the context of the nonparametric kernel function, least-squares cross-validation, plug-in, and cross-validation procedures were considered for bandwidth selection. Overall, all the methods performed well both theoretically and practically; however, the least-squares cross-validation method needs a very large sample size to achieve satisfactory findings [58,59]. The two different plug-in bandwidth approaches were investigated by [58,60]. The cross-validation method was developed by [61], which showed promising results. Further, it was discovered that both cross-validation and plug-in bandwidths produced similar results in terms of DF estimation, but cross-validation had a clear disadvantage in terms of computation time [53,54,59]. The plug-in method, which has been employed previously in similar studies, yielded excellent results [54,60,62]. We used the plug-in approach suggested by [60] to determine the bandwidth for nonparametric kernel estimation of the DF of APFDs in this framework. The interested reader can obtain more theoretical information and a comprehensive explanation of the Polansky and Baker plug-in approach for bandwidth selection of the nonparametric DF; for details, see [59,60].

## 3. Study Area and Data

Climate change has significantly impacted the entire world. Its impacts may vary from increases in the magnitude and frequency of natural disasters, such as floods and droughts, to the extinction of species and the spread of vector-borne diseases. However, the effects of climate change are not equally observed across the globe. As a matter of fact, developing countries are much more vulnerable to climate change-related hazards, mainly due to their lack of proper infrastructure. For instance, Pakistan has suffered significant economic losses in the past 6 to 7 years as a result of the recent increases on the melting rate of South Asia’s glaciers, which leads to more frequent and severe floods. 

**Figure 1:**
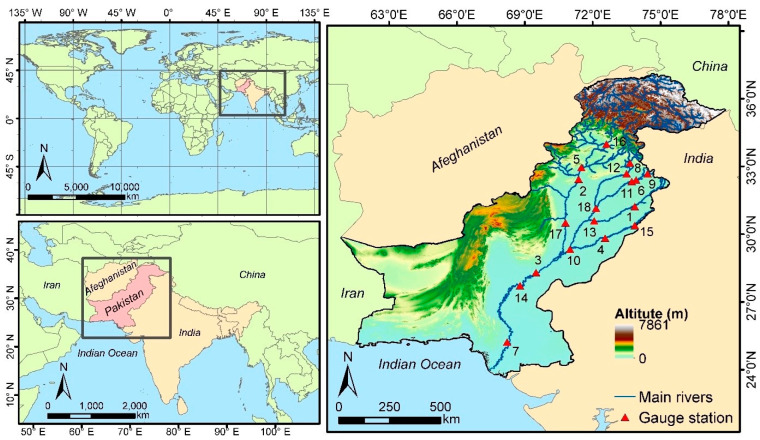
Geographical locations of the eighteen sites of Punjab, Pakistan, used in this study.

Pakistan has a population of around 208 million people encompassing an area of approximately 796,000 km^2^. The country is bounded by the Himalayan Mountains to the north, India to the east, Iran to the west, and the Indian Ocean to the south (Figure 1). Its altitudes vary from 8500 m in the northern regions to 0 m in the coastal regions, thus having a strong orographic influence over monsoon winds. Pakistan’s climate is usually considered hot and dry, being classified as semi-arid in the south and dry cold in the north by means of Koppen climate classes [63]. 

The Indus River and its tributaries, i.e., Sutlej, Beas, Ravi, Chenab, Jhelum, Swat, and Kabul, are vital to the economy of Pakistan, as they are the main source of water for irrigation, industry, and urban water supply. However, this river network is also responsible for economic losses through large flood events, most frequent in the Punjab and Sindh regions, affecting not only fertile agricultural lands, but also large urban centers near the river network. This situation is aggravated when considering future climate change scenarios, as higher variabilities in precipitation and glacier melting are projected, as well as rises in sea level and storm surges, leading to stress of current drainage network systems, especially during the monsoon season.

In this study, the annual maximum peak flows data of 18 sites of Pakistan, located on five rivers, namely, Indus, Jhelum, Chenab, Ravi, and Sutlej, were used in this study. The geographical location of these river sites is given in Figure 1. The data for these sites were collected from the hydrology department of the Water and Power Development Authority (WAPDA) and the Federal Flood Commission. Most of the annual maximum peak flows at sites were recorded in the peak of the monsoon season (from July to September). The annual peak flow discharge of 18 stations was measured in cusec. Summary statistics for 18 stations are given in Table 1. The highest mean peak flow discharge was recorded at the Guddu station (609,909.423), and the lowest mean peak flow discharge was observed at Islam station (49,089.45). The highest standard deviations were observed at Kotri, Sukkur, Guddu, Qadirabad, Khanki, and Trimmu stations. The range of skewness varied from 0.552 to 4.240, while Guddu had the smallest skewness, and Mangla had the largest skewness. Similarly, the kurtosis varied from −0.645 to 22.770, with Mangle, Kotri, Rasul, and Tarbela having the highest Kurtosis, while the lowest kurtosis was observed at Sukkur, Guddu, Qadirabad, and Marala stations. 

## 4. Results and Discussion

The AD test, KS test, and CVM test were applied to each station in order to choose the best-fit PDF among the GEV, GLO, and GPA. The selection of the best fitted PDF for each station was based on the smallest GOF tests among the three PDFs. The best fitted PDF results for each station according to GOF tests at a 5% significance level are reported in Table 2.

In the case of η = 0, it is clear from Table 2 that GPA and GEV distributions were best fitted for seven stations, while GLO was best fitted for four stations according to the AD test. Similarly, the KS test results well matching the results of the AD test except for Taunsa, Guddu, Khanki, and Panjnad stations. According to the KS test results for η = 0, the most appropriate PDFs were GPA and GEV. Investigating the CVM test results for η = 0, the GEV distribution was suitable for most stations, followed by GPA distribution, which was selected for six stations. Overall, in the case of η = 0, the results of the AD test, KS test, and CVM test indicated that the GEV and GPA distributions were the most adequate for most stations. 

Moving forward to η = 1 in Table 2, the AD test selected the GEV distribution for the highest number of stations (10 stations out of 18) followed by GPA distribution, which was selected for five stations. However, the KS test and CVM test selected the GEV and GPA distributions for the same number of stations, 8 and 7, respectively. Considering AD test, KS test, and CVM test results for η = 1, the GEV distribution was selected for eight stations, the GPA distribution for seven stations, and the GLO distribution for only three stations. Finally, for η = 2, it is observed from Table 2 that the AD test and CVM test selected GPA distribution for eight stations, GEV distribution for seven stations, and GLO distribution for the remaining four stations. On the other hand, GPA and GEV distributions each yielded the best-fit for seven stations based on the KS test. 

It is also worthwhile to mention that the GOF test produced different results for Mangla, Rasul, and Panjnad stations as we increased the value η = 1, 2. However, in general, increasing η = 1, 2 had no effect on the results for most stations.

**Figure 2:**
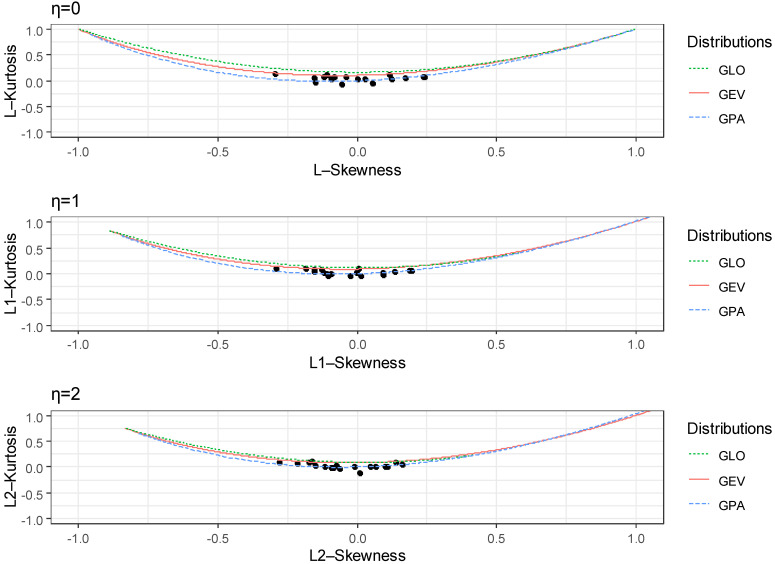
LH-ratio diagram for 18 stations.

The LH-ratio diagram is a useful tool that simplifies analysis, demonstrating the versatility of how various PDFs plot. Furthermore, it can be shown that PDFs can have several different skewness and kurtosis values, rendering them more valuable for analyzing the shape of the distribution. The LH-ratio diagram for η = 0, η = 1, and η = 2 of 18 stations was plotted in Figure 2. It is observed from Figure 2, for η = 0, that most of the scatter points were between the GEV and GPA distributions curves, whereas a few scattered points were closed to the GLO distribution curve. Therefore, according to the L-ratio diagram (η = 0), GEV and GPA were the most suitable PDFs for the annual peak flow series of 18 stations. By observing Figure 2 in the case of η = 1 and η = 2, most of scattered points were closed to the GPA distribution curve, followed by GEV. We also note from Figure 2 that as we increased η = 1, 2, peak flow series tended to follow GPA and GEV distributions. Overall, the findings obtained from GOF tests were generally in good agreement with the LH-ratio diagram for most stations.

**Figure 3:**
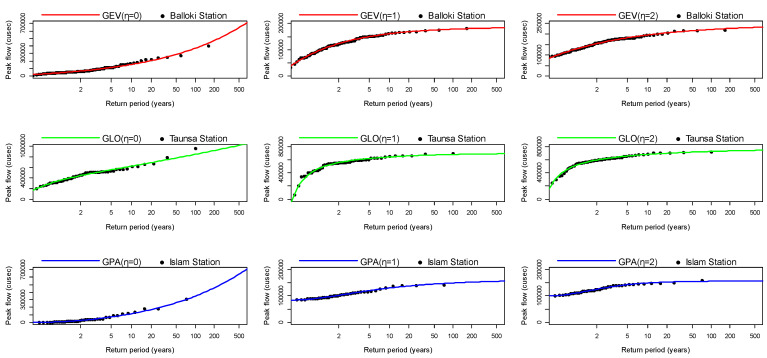
Relation between return period and annual peak flow based on the LH-moments.

Further the relation between the return period and APFD was also established for GEV, GLO, and GPA distributions. Figure 3 shows the curves for Balloki, Taunsa, and Islam stations, highlighting how well the APFD series at lower return periods and upper return periods were estimated by LH-moments (η = 0, 1, 2). It is seen from Figure 3 that the GEV, GPA, and GLO distributions well fitted the observed APFD series at lower and higher return periods. Figure 3 indicates that as the level of LH-moments increased (η = 1, 2), the GEV, GLO, and GPA distributions performed well in reflecting the extreme tail at higher return periods. In Figure 3, it is noted that most of the observations fell within 2–50 years (0.02 ≤ *p* ≤ 0.5), implying that hazardous flood events with low probability or large return periods (100 and 500) have rarely occurred at these stations.

**Figure 4:**
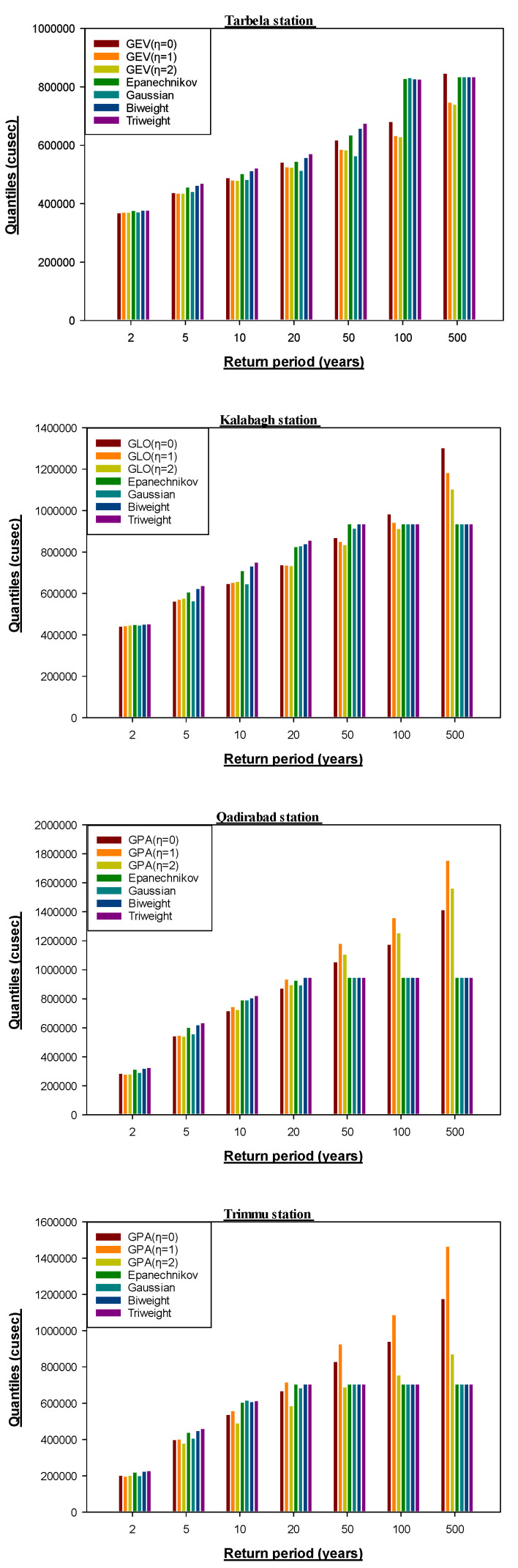
Quantiles for best-fit PDFs and nonparametric kernel functions for four randomly selected stations.

In the planning and design of hydrological systems, it is critical to determine the return period since a given flood event. Further, we calculated quantiles for different return periods using LH-moments (η = 0, 1, 2) and nonparametric kernel functions; results for Tarbela, Kalabagh, Qadirabad, and Trimmu stations are reported in Figure 4. The results of quantile estimates in Figure 4 can be interpreted as follows: for Tarbela station’s return period of 500 years, the GEV distribution (η = 0) produced quantile (847,553.3) is the threshold value of flow that may occur once every 500 years on average. In other words, there is only a 0.2% chance that in a return period of 500 years, one-time discharge (peak flow) will exceed the threshold value (847,553.3) and consequently a flood will occur. At the same time, 99.9% is the chance that the one-time discharge (peak flow) will be less than the threshold value (847,553.3) in a return period of 500 years. 

We also investigated the impact of LH-moment choice (η = 0, 1, 2) and nonparametric kernel functions on estimating quantiles associated with predefined return periods via relative absolute error (RAE). The RAE is an assessment of the difference between the actual flood estimate and the flood estimate by the best-fit PDF. The RAE was calculated using the following equation, described in [35,48,64].

(50) $$RAE=\left|\frac{X-Y}{Y}\right|\;$$

where *X* is the actual peak flow, and *Y* denotes the design quantile estimate obtained through the LH-moments and the nonparametric kernel function. 

Table 3 and Table 4 summarize the RAE associated with each station using LH-moments and a nonparametric kernel function, emphasizing the significance of evaluating techniques for diverse return periods. It is crucial to investigate the discrepancy between actual flood estimates and quantiles produced via PDF and the nonparametric kernel function. Although all these PDFs passed GOF testing, there were still considerable discrepancies in quantile estimations. These discrepancies are significant to policymakers, planners, and decision-makers.

As shown in Table 3, the GLO, GEV, and GPA distributions produced very small errors for all stations. In Table 3, the results of GEV distribution for Tarbela, Guddu, Kotri, Khanki, Balloki, and Sidhani stations based on L2-moment (η = 2) produced a small error for all return periods. However, it was noticed that for return periods of 5 years, the GEV distribution provided the same result for Kotri and Guddu stations based on L1-moment (η = 1) and L2-moment (η = 2). 

According to Table 3, the RAE findings for return periods (2, 5, and 10 years) for GLO distribution based on L-moment (η = 0), L1-moment (η = 1) and L2-moment (η = 2) for Kalabagh, Chashma, and Taunsa stations had a fairly close error, whereas for the remaining return periods, L2-moment (η = 2) had a clear edge over the L-moment (η = 0) and L1-moment (η = 1). It was also observed, as seen in Table 3, that the GPA distribution using L2-moment (η = 2) produced a minimal error for all return periods for Sukkur, Marala, Qadirabad, Sulemanki, and Islam stations. 

Table 3 indicates that for Mangla station, the RAE for GLO and GEV distributions were estimated through L-moment (η = 0), L2-moment (η = 2) and L1-moment (η = 1), respectively; however, the GLO and GEV distributions produced the same amount of error for the return periods (2, 5 and 10 years) when using the L2-moment (η = 2) and L1-moment (η = 1), but the L2-moment (η = 2) had the edge for return the periods of 20, 50, 100, and 500 years. Similarly, the findings for Rasul and Panjnad stations indicated that GPA distribution using L2-moment (η = 2) had lower values of RAE at all return periods than did GEV distribution when using L-moment (η = 0) and L1-moment (η = 1).

Moreover, as can be seen in Table 3, the findings of RAE for all stations using L-moment (η = 0), L1-moment (η = 1), and L2-moment (η = 2) at the return periods of (2, 5, 10, 20 years) were close, but L1-moment (η = 1) and L2-moment (η = 2) had a slight advantage over L-moment (η = 0). However, for high return periods (50, 100, 500 years), the L2-moment (η = 2) performed better than the L1-moment (η = 1) and L-moment (η = 0). We also observed, as seen in Table 3, that with the increasing level of LH-moments (η = 0, 1, 2), the error became smaller, especially for high return periods. In other words, the L2-moment (η = 2) yielded the lowest error compared to the L1-moment (η = 1) and L-moment (η = 0). Additionally, it was found that there were a few overlaps among these PDFs for certain return periods. For example, for the Mangla station at return periods of 2, 5, and 10 years, the GEV and GLO distributions yielded the same amount of error (0.016, 0.018, and 0.026). This implies that the performance of the PDFs was the same for a certain return period.

Table 4 compares quantile estimates in terms of RAE for Epanechnikov, Gaussian, Biweight, and Triweight kernel functions for all stations. It is evident from Table 4 that the Gaussian kernel function had the lowest RAE throughout all return periods among the Epanechnikov, Biweight, and Triweight kernel functions for Tarbela, Kalabagh, Taunsa, Guddu, Sukkur, Kotri, Mangla, Rasul, Marala, Khanki, Qadirabad, Trimmu, Sidhani, Sulemanki, and Islam stations. 

The results in Table 4 for Chashma station indicate that the Triweight kernel function had a low RAE for the return period of 2 years, followed by the Biweight and Epanechnikov kernel functions; however, the Gaussian kernel function performed better for the return periods of 5, 10, 20, 50, 100, and 500 years. Similarly, the findings for the Balloki station revealed that the Biweight kernel function had an edge at the return periods of 2 and 5 years. Additionally, we also observed that the Epanechnikov kernel function had a lower RAE than Gaussian, Biweight, and Triweight kernel functions for the Panjnad station at a return period of 2 years, whereas the Epanechnikov, Biweight, and Triweight kernel functions performed equally well for the return period of 5 years. In accordance with the findings of the previous study [55], the results of our investigation demonstrated that among nonparametric kernel functions, the Gaussian kernel function performed best for the observed flood.

Finally, to evaluate the performance of the LH-moments (η = 0, 1, 2), the nonparametric kernel functions in terms of the RAE measures of quantile estimates were calculated and given in Table 3 and Table 4. The findings reveal that the LH-moments (η = 0, 1, 2) led to more accurate estimates for most of the stations than did the nonparametric kernel function. On the other hand, it was also noted that the nonparametric kernel function performed better than LH-moments (η = 0, 1, 2) for Kalabagh, Chashma, and Guddu stations at return periods of 2, 5, and 10 years. Besides that, among nonparametric kernel functions, the Gaussian kernel function provides very close estimates for smaller return periods as compared to LH-moments (η = 0). Similar findings were reported by Adamowski et al. [52], who evaluated L-moments and nonparametric methods for the annual maxima and partial duration flood series. However, in the case of L2-moments (η = 2), we found significant differences, specifically in the higher quantile estimates, with nonparametric kernel functions. This ensures that the L2-moments (η = 2) accurately estimate the extreme quantiles for the current dataset compared to any other approach considered in this work.

## 5. Conclusions

Estimating quantiles is a widespread practice in hydrology, and it is often used in the planning, design, and operation of a hydraulic system. In this study, we employed LH-moments (η = 0, 1, 2) and a nonparametric kernel function to estimate the peak flow series at 18 stations in Punjab, Pakistan. Based on the findings of this study, the following conclusions may be drawn: 

**Main findings of the paper:** The findings of the AD test, KS test, CVM test, and LH-ratio diagram indicate that the best fits PDFs for estimating peak-flow data are GPA, followed by GEV and GLO distributions. It was identified that by raising the value of (η = 1, 2) in the LH-moments, the GOF test produced different findings for Mangla, Rasul, and Panjnad stations; nevertheless, increasing (η = 1, 2) did not affect the results for the rest of the stations. The magnitudes of quantile estimates obtained using the nonparametric kernel function are greater than those obtained through LH-moments (η = 0, 1, 2). Overall, the LH-moments (η = 0, 1, 2) accurately estimate the quantile in terms of RAE for most of the stations; however, for Kalabagh, Chashma, and Guddu stations at return periods of 2, 5, and 10 years, nonparametric kernel function provide smaller RAE than LH-moments (η = 0, 1, 2). The L-moment (η = 0), L1-moment (η = 1), and L2-moment (η = 2) provide relatively close estimates of quantile errors for all stations at the return periods of 2, 5, 10, and 20 years; moreover, L2-moments (η = 2) yielded the lowest error for the higher return period of 50, 100, and 500 years among L-moment (η = 0), L1-moments (η = 1), and nonparametric kernel functions. We also found that among nonparametric kernel functions for small return periods, the Gaussian kernel function provides a very close estimate compared to LH-moments (η = 0, 1, 2).

**Limitations of this work and future research:** Further research is needed on nonparametric kernel functions, specifically for large return periods, to improve the results in terms of RAE. This is the first application of nonparametric kernel functions in flood frequency analysis of 18 stations in Punjab, Pakistan. This research may be expanded by integrating all of Pakistan’s river gauging stations in order to determine the best estimation methods for the whole country.

**Broader impacts:** The findings of this research will enhance recommendations for future development to preserve current infrastructure and minimize economic damage due to floods. Additionally, the findings will also aid in designing and implementing flood mitigation measures, such as more effective stormwater management.

## Figures and Tables

**Table 1:** Summary statistics of 18 stations.

Name of Stations	River	Latitude (North)	Longitude (East)	Period (Years)	Mean	Standard Deviation	Skewness	Kurtosis	Minimum Peak Flow	Maximum Peak Flow
Tarbela	Indus	33.99	72.61	1960–2013	386,962.963	87,785.537	2.626	11.806	273,000	835,000
Kalabagh	Indus	32.95	71.50	1968–2013	464,719.956	151,843.363	1.186	2.102	237,297	936,453
Chashma	Indus	32.43	71.38	1971–2013	475,333.046	149,635.274	1.22	3.727	214,045	1,038,873
Taunsa	Indus	30.50	70.80	1958–2013	452,791.554	140,793.102	0.804	2.144	182,372	959,991
Guddu	Indus	28.30	69.50	1962–2013	609,909.423	284,534.413	0.552	−0.557	170,831	1,176,150
Sukkur	Indus	27.72	68.79	1982–2013	546,609.594	309,470.519	0.629	−0.645	126,130	1,172,000
Kotri	Indus	25.22	68.22	1970–2013	395,262.068	379,599.333	3.705	18.290	47,100	2,409,000
Mangla	Jhelum	33.15	73.65	1960–2013	132,481.778	136,385.297	4.240	22.770	20,460	932,700
Rasul	Jhelum	32.68	73.50	1970–2013	134,418.386	161,219.596	3.582	15.787	19,702	952,170
Marala	Chenab	32.68	74.43	1960–2013	308,572.407	196,419.272	1.097	0.227	93,150	792,765
Khanki	Chenab	32.40	73.92	1925–2013	351,963.191	242,710.633	1.494	1.391	97,058	1,086,460
Qadirabad	Chenab	32.33	73.73	1970–2013	356,547.704	247,771.998	1.030	0.106	76,336	948,520
Trimmu	Chenab	31.14	72.15	1968–2013	261,376.217	194,828.961	1.099	0.1693	42,756	706,433
Panjnad	Chenab	29.33	71.00	1960–2013	260,134.722	193,661.339	0.980	0.554	17,833	802,516
Balloki	Ravi	31.22	73.86	1922–2013	87,914.728	64,039.572	2.183	6.180	14,000	399,356
Sidhani	Ravi	30.58	72.07	1925–2013	64,143.427	56,691.878	2.159	4.916	8488	296,086
Sulemanki	Sutlej	30.38	73.86	1975–2013	70,254.923	84,914.177	2.267	5.865	1506	399,453
Islam	Sutlej	29.82	72.55	1974–2013	49,089.45	63,209.754	2.362	6.497	1231	306,425

**Table 2:** GOF results for GEV, GLO, and GPA distributions using LH-moments (η = 0, 1, 2).

Stations	L-Moments (η = 0)			L1-Moments (η = 1)			L2-Moments (η = 2)		
	AD Test	KS Test	CVM Test	AD Test	KS Test	CVM Test	AD Test	KS Test	CVM Test
Tarbela	GEV(0.984)	GEV(0.975)	GEV(0.973)	GEV(0.395)	GEV(0.605)	GEV(0.670)	GEV(0.233)	GEV(0.332)	GLO(0.360)
Kalabagh	GLO(0.954)	GLO(0.938)	GLO(0.972)	GLO(0.855)	GLO(0.785)	GLO(0.876)	GLO(0.642)	GLO(0.682)	GLO(0.696)
Chashma	GLO(0.983)	GLO(0.965)	GLO(0.970)	GLO(0.943)	GLO(0.895)	GLO(0.951)	GLO(0.801)	GLO(0.836)	GLO(0.853)
Taunsa	GLO(0.811)	GEV(0.537)	GLO(0.705)	GLO(0.832)	GEV(0.606)	GLO(0.761)	GLO(0.762)	GLO(0.627)	GLO(0.753)
Guddu	GEV(0.740)	GLO(0.878)	GEV(0.769)	GEV(0.765)	GLO(0.889)	GEV(0.781)	GEV(0.742)	GEV(0.923)	GEV(0.790)
Sukkur	GPA(0.990)	GPA(0.978)	GPA(0.992)	GPA(0.944)	GPA(0.962)	GPA(0.960)	GPA(0.963)	GPA(0.988)	GPA(0.971)
Kotri	GEV(0.974)	GEV(0.837)	GEV(0.924)	GEV(0.947)	GEV(0.821)	GEV(0.916)	GEV(0.859)	GEV(0.831)	GLO(0.887)
Mangla	GLO(0.956)	GLO(0.900)	GLO(0.932)	GEV(0.803)	GEV(0.864)	GEV(0.916)	GEV(0.537)	GLO(0.851)	GLO(0.877)
Rasul	GEV(0.946)	GEV(0.984)	GLO(0.939)	GEV(0.962)	GEV(0.988)	GEV(0.950)	GPA(0.928)	GEV(0.991)	GPA(0.943)
Marala	GPA(0.969)	GPA(0.973)	GPA(0.974)	GPA(0.758)	GPA(0.823)	GPA(0.880)	GPA(0.735)	GPA(0.875)	GPA(0.787)
Khanki	GEV(0.693)	GPA(0.868)	GEV(0.744)	GEV(0.612)	GEV(0.713)	GEV(0.712)	GEV(0.465)	GEV(0.741)	GEV(0.655)
Qadirabad	GPA(0.995)	GPA(0.996)	GPA(0.999)	GEV(0.930)	GPA(0.983)	GPA(0.988)	GPA(0.943)	GPA(0.985)	GPA(0.968)
Trimmu	GPA(0.779)	GPA(0.778)	GPA(0.726)	GEV(0.683)	GPA(0.679)	GPA(0.699)	GEV(0.698)	GPA(0.622)	GPA(0.648)
Panjnad	GPA(0.908)	GEV(0.879)	GEV(0.878)	GPA(0.914)	GPA(0.885)	GPA(0.894)	GPA(0.933)	GPA(0.897)	GPA(0.906)
Balloki	GEV(0.582)	GEV(0.624)	GEV(0.551)	GEV(0.486)	GEV(0.621)	GEV(0.517)	GEV(0.325)	GEV(0.621)	GEV(0.480)
Sidhani	GEV(0.978)	GEV(0.990)	GEV(0.974)	GEV(0.971)	GEV(0.992)	GEV(0.969)	GEV(0.933)	GEV(0.982)	GEV(0.957)
Sulemanki	GPA(0.996)	GPA(0.994)	GPA(0.998)	GPA(0.998)	GPA(0.991)	GPA(0.998)	GPA(0.999)	GPA(0.990)	GPA(0.998)
Islam	GPA(0.900)	GPA(0.753)	GPA(0.877)	GPA(0.931)	GPA(0.693)	GPA(0.885)	GPA(0.936)	GPA(0.715)	GPA(0.886)

**Table 3:** RAE of quantile estimates for GEV, GLO, and GPA distributions using LH-moments (η = 0, 1, 2).

Station Name	Best Fitted Distribution	2	5	10	20	50	100	500
Tarbela	(η = 0)	0.008	0.009	0.015	0.028	0.053	0.077	0.156
	GEV (η = 1)	0.004	0.005	0.008	0.011	0.017	0.022	0.035
	(η = 2)	0.003	0.004	0.006	0.008	0.011	0.014	0.02
Kalabagh	(η = 0)	0.011	0.012	0.023	0.041	0.072	0.103	0.202
	GLO (η = 1)	0.009	0.012	0.016	0.021	0.03	0.039	0.063
	(η = 2)	0.009	0.013	0.015	0.018	0.024	0.029	0.045
Chashma	(η = 0)	0.01	0.012	0.022	0.036	0.061	0.085	0.156
	GLO (η = 1)	0.01	0.014	0.017	0.022	0.03	0.037	0.059
	(η = 2)	0.011	0.014	0.016	0.019	0.025	0.031	0.048
Taunsa	(η = 0)	0.009	0.012	0.019	0.029	0.046	0.061	0.106
	GLO (η = 1)	0.01	0.014	0.016	0.02	0.027	0.033	0.05
	(η = 2)	0.01	0.013	0.015	0.019	0.025	0.03	0.046
Guddu	(η = 0)	0.016	0.016	0.025	0.041	0.065	0.087	0.144
	GEV (η = 1)	0.013	0.015	0.022	0.032	0.047	0.06	0.09
	(η = 2)	0.012	0.015	0.02	0.028	0.039	0.047	0.067
Sukkur	(η = 0)	0.023	0.026	0.032	0.053	0.086	0.112	0.172
	GPA (η = 1)	0.021	0.024	0.03	0.048	0.076	0.096	0.14
	(η = 2)	0.018	0.021	0.027	0.043	0.066	0.082	0.114
Kotri	(η = 0)	0.03	0.041	0.043	0.081	0.159	0.235	0.483
	GEV (η = 1)	0.019	0.021	0.03	0.047	0.075	0.099	0.163
	(η = 2)	0.017	0.021	0.028	0.039	0.054	0.067	0.096
Mangla	GLO (η = 0)	0.042	0.046	0.073	0.079	0.159	0.235	0.47
	GEV (η = 1)	0.016	0.018	0.026	0.042	0.068	0.09	0.148
	GLO (η = 2)	0.016	0.018	0.026	0.037	0.056	0.072	0.119
Rasul	GEV (η = 0)	0.056	0.067	0.089	0.096	0.174	0.256	0.505
	GEV (η = 1)	0.022	0.034	0.036	0.065	0.113	0.157	0.285
	GPA (η = 2)	0.018	0.022	0.027	0.044	0.069	0.087	0.124
Marala	(η = 0)	0.022	0.024	0.031	0.052	0.09	0.124	0.214
	GPA (η = 1)	0.017	0.018	0.025	0.041	0.069	0.092	0.151
	(η = 2)	0.013	0.014	0.019	0.031	0.049	0.063	0.094
Khanki	(η = 0)	0.021	0.026	0.036	0.056	0.112	0.166	0.337
	GEV (η = 1)	0.012	0.017	0.021	0.04	0.073	0.103	0.189
	(η = 2)	0.009	0.01	0.016	0.025	0.04	0.053	0.087
Qadirabad	(η = 0)	0.025	0.029	0.034	0.057	0.098	0.133	0.226
	GPA (η = 1)	0.022	0.023	0.029	0.048	0.079	0.105	0.166
	(η = 2)	0.018	0.019	0.025	0.041	0.065	0.082	0.119
Trimmu	(η = 0)	0.027	0.032	0.035	0.06	0.105	0.145	0.253
	GPA (η = 1)	0.022	0.024	0.03	0.05	0.083	0.109	0.175
	(η = 2)	0.017	0.02	0.025	0.042	0.064	0.079	0.109
Panjnad	GEV (η = 0)	0.02	0.031	0.035	0.058	0.099	0.135	0.235
	GPA (η = 1)	0.018	0.026	0.03	0.048	0.071	0.086	0.112
	GPA (η = 2)	0.015	0.022	0.026	0.044	0.068	0.079	0.096
Balloki	(η = 0)	0.023	0.026	0.038	0.056	0.114	0.17	0.343
	GEV (η = 1)	0.011	0.014	0.02	0.035	0.061	0.084	0.149
	(η = 2)	0.008	0.01	0.014	0.021	0.032	0.04	0.06
Sidhani	(η = 0)	0.028	0.028	0.047	0.06	0.123	0.182	0.362
	GEV (η = 1)	0.013	0.02	0.023	0.042	0.073	0.099	0.173
	(η = 2)	0.012	0.012	0.019	0.03	0.046	0.059	0.093
Sulemanki	(η = 0)	0.053	0.059	0.085	0.09	0.173	0.253	0.512
	GPA (η = 1)	0.037	0.042	0.054	0.076	0.138	0.193	0.355
	(η = 2)	0.029	0.039	0.044	0.069	0.118	0.158	0.263
Islam	(η = 0)	0.058	0.068	0.091	0.096	0.175	0.256	0.518
	GPA (η = 1)	0.042	0.044	0.063	0.08	0.149	0.211	0.397
	(η = 2)	0.031	0.039	0.046	0.07	0.121	0.164	0.278

**Table 4:** RAE of quantile estimates for Epanechnikov, Gaussian, Biweight, and Triweight kernel functions.

Station Name	Kernel Function Type	2	5	10	20	50	100	500
Tarbela	Epanechnikov	0.027	0.033	0.046	0.064	0.182	0.346	0.728
	Gaussian	0.014	0.02	0.027	0.049	0.079	0.12	0.24
	Biweight	0.029	0.047	0.064	0.087	0.211	0.39	0.74
	Triweight	0.03	0.06	0.081	0.107	0.23	0.31	0.5
Kalabagh	Epanechnikov	0.01	0.024	0.101	0.124	0.2	0.23	0.33
	Gaussian	0.004	0.01	0.018	0.023	0.032	0.07	0.125
	Biweight	0.006	0.013	0.125	0.127	0.14	0.19	0.21
	Triweight	0.014	0.016	0.145	0.149	0.17	0.198	0.24
Chashma	Epanechnikov	0.004	0.078	0.081	0.12	0.183	0.263	0.58
	Gaussian	0.005	0.01	0.024	0.068	0.088	0.2	0.534
	Biweight	0.003	0.055	0.127	0.152	0.214	0.434	0.63
	Triweight	0.002	0.029	0.128	0.178	0.239	0.488	0.678
Taunsa	Epanechnikov	0.019	0.089	0.097	0.101	0.103	0.121	0.2
	Gaussian	0.014	0.017	0.02	0.025	0.046	0.067	0.167
	Biweight	0.019	0.115	0.116	0.122	0.129	0.222	0.29
	Triweight	0.019	0.134	0.139	0.14	0.153	0.267	0.32
Guddu	Epanechnikov	0.013	0.014	0.023	0.091	0.182	0.311	0.671
	Gaussian	0.003	0.005	0.017	0.042	0.11	0.224	0.422
	Biweight	0.02	0.023	0.033	0.11	0.196	0.375	0.76
	Triweight	0.025	0.023	0.054	0.127	0.215	0.46	0.845
Sukkur	Epanechnikov	0.032	0.035	0.069	0.139	0.216	0.297	0.532
	Gaussian	0.027	0.03	0.035	0.06	0.1	0.19	0.383
	Biweight	0.035	0.041	0.089	0.171	0.342	0.441	0.72
	Triweight	0.035	0.046	0.089	0.199	0.438	0.564	0.783
Kotri	Epanechnikov	0.095	0.131	0.162	0.191	0.257	0.501	0.732
	Gaussian	0.04	0.055	0.083	0.15	0.2	0.295	0.527
	Biweight	0.05	0.11	0.13	0.158	0.222	0.45	0.69
	Triweight	0.073	0.124	0.15	0.181	0.245	0.489	0.705
Mangla	Epanechnikov	0.107	0.127	0.132	0.154	0.231	0.476	0.845
	Gaussian	0.051	0.068	0.099	0.134	0.198	0.345	0.695
	Biweight	0.12	0.153	0.183	0.203	0.282	0.523	0.912
	Triweight	0.135	0.17	0.185	0.212	0.292	0.545	0.989
Rasul	Epanechnikov	0.069	0.092	0.105	0.15	0.315	0.605	1.21
	Gaussian	0.06	0.09	0.099	0.13	0.265	0.55	0.999
	Biweight	0.083	0.101	0.163	0.193	0.386	0.71	1.421
	Triweight	0.085	0.105	0.183	0.213	0.412	0.8	1.89
Marala	Epanechnikov	0.03	0.045	0.055	0.08	0.12	0.223	0.525
	Gaussian	0.028	0.04	0.053	0.069	0.1	0.193	0.412
	Biweight	0.055	0.075	0.097	0.13	0.274	0.498	0.875
	Triweight	0.067	0.091	0.104	0.198	0.32	0.53	0.995
Khanki	Epanechnikov	0.081	0.09	0.124	0.175	0.243	0.475	0.822
	Gaussian	0.043	0.075	0.106	0.135	0.203	0.422	0.79
	Biweight	0.099	0.109	0.141	0.19	0.275	0.49	0.918
	Triweight	0.103	0.116	0.142	0.203	0.303	0.503	1.116
Qadirabad	Epanechnikov	0.046	0.061	0.076	0.122	0.17	0.328	0.631
	Gaussian	0.029	0.052	0.076	0.105	0.152	0.298	0.608
	Biweight	0.058	0.073	0.079	0.139	0.185	0.347	0.675
	Triweight	0.063	0.079	0.096	0.151	0.196	0.365	0.692
Trimmu	Epanechnikov	0.038	0.083	0.108	0.244	0.331	0.644	1.976
	Gaussian	0.025	0.058	0.101	0.175	0.305	0.563	1.107
	Biweight	0.033	0.063	0.106	0.261	0.36	0.682	2.19
	Triweight	0.033	0.073	0.125	0.273	0.36	0.705	2.806
Panjnad	Epanechnikov	0.034	0.073	0.095	0.109	0.136	0.275	0.595
	Gaussian	0.047	0.057	0.083	0.105	0.126	0.234	0.498
	Biweight	0.038	0.073	0.117	0.128	0.143	0.283	0.607
	Triweight	0.064	0.073	0.126	0.156	0.17	0.303	0.67
Balloki	Epanechnikov	0.031	0.047	0.077	0.119	0.177	0.219	0.445
	Gaussian	0.04	0.049	0.064	0.108	0.133	0.212	0.414
	Biweight	0.028	0.043	0.092	0.124	0.192	0.324	0.59
	Triweight	0.029	0.046	0.105	0.135	0.205	0.335	0.67
Sidhani	Epanechnikov	0.068	0.081	0.095	0.155	0.218	0.402	0.851
	Gaussian	0.041	0.051	0.075	0.131	0.197	0.359	0.738
	Biweight	0.085	0.096	0.105	0.174	0.29	0.507	0.907
	Triweight	0.098	0.104	0.118	0.192	0.317	0.541	0.942
Sulemanki	Epanechnikov	0.068	0.112	0.146	0.182	0.268	0.573	1.165
	Gaussian	0.063	0.078	0.112	0.148	0.233	0.438	0.91
	Biweight	0.071	0.087	0.137	0.185	0.271	0.518	1.154
	Triweight	0.071	0.082	0.132	0.155	0.251	0.502	1.123
Islam	Epanechnikov	0.099	0.145	0.191	0.245	0.399	0.745	1.168
	Gaussian	0.067	0.11	0.154	0.21	0.367	0.61	0.929
	Biweight	0.109	0.168	0.217	0.268	0.409	0.778	1.481
	Triweight	0.118	0.19	0.26	0.329	0.418	0.819	1.921
